# CRISPR/Cas9: a powerful tool in colorectal cancer research

**DOI:** 10.1186/s13046-023-02901-z

**Published:** 2023-11-22

**Authors:** Yang Hu, Liang Liu, Qi Jiang, Weiping Fang, Yazhu Chen, Yuntian Hong, Xiang Zhai

**Affiliations:** 1 Department of Gastroenterology, The First People’s Hospital of Jiande, Hangzhou, 311600 China; 2 Department of Orthopedic Surgery, Zhongnan Hospital of Wuhan University, Wuhan, 430071 China; 3 Department of Biological Repositories, Zhongnan Hospital of Wuhan University, Wuhan, 430071 China; 4 West China Hospital of Sichuan University, Chengdu, 610044 China; 5 Department of Gastroenterology, Zhongnan Hospital of Wuhan University, Wuhan, 430071 China; 6 Department of Colorectal and Anal Surgery, Zhongnan Hospital of Wuhan University, Wuhan, 430071 China

**Keywords:** CRISPR/Cas9, CRC, Gene editing tool, Target therapy, Precise medicine, Off-target

## Abstract

Colorectal cancer (CRC) is one of the most common malignant cancers worldwide and seriously threatens human health. The clustered regulatory interspaced short palindromic repeat/CRISPR-associate nuclease 9 (CRISPR/Cas9) system is an adaptive immune system of bacteria or archaea. Since its introduction, research into various aspects of treatment approaches for CRC has been accelerated, including investigation of the oncogenes, tumor suppressor genes (TSGs), drug resistance genes, target genes, mouse model construction, and especially in genome-wide library screening. Furthermore, the CRISPR/Cas9 system can be utilized for gene therapy for CRC, specifically involving in the molecular targeted drug delivery or targeted knockout in vivo. In this review, we elucidate the mechanism of the CRISPR/Cas9 system and its comprehensive applications in CRC. Additionally, we discussed the issue of off-target effects associated with CRISPR/Cas9, which serves to restrict its practical application. Future research on CRC should in-depth and systematically utilize the CRISPR/Cas9 system thereby achieving clinical practice.

## Introduction

Colorectal cancer (CRC) is one of the most common malignant cancers worldwide, with 1,931,590 cases diagnosed globally in 2020 [1]. Moreover, morbidity and mortality of CRC have increased in recent years (rank 3d and 2d, respectively), thus aggravating the economic burden and affecting public health [2, 3]. Genetic aberrations such as RAS activation, APC mutation, and TP53 loss of function have been reported to be involved in most CRCs. Moreover, mutS homologs (*MSH2, MSH3, MSH4, MSH5, MSH6*) and mutL homologs (*MLH1, MLH3*) mutation can also lead to microsatellite instable cancers [4, 5]. Despite the significant progress in various treatment regimens such as cytotoxic chemotherapy, molecular-targeted therapy, and immunotherapy, the 5-year survival rate of advanced CRC merely improved (survival rate of the individuals during 2000 to 2014 that ages over 50 decreased 34%, but increased 13% in those ages less than 50 years) [6]. One major factor is that 90% of CRC patients have congenital or acquired drug resistance [7]. Meanwhile, due to non-high levels of microsatellite instability (non-MSI-H) and proficient mismatch repair (pMMR) CRCs do not respond to PD-1/PD-L1 inhibitors [8]. Therefore, investigating new biological mechanisms and hallmarks of CRC for early diagnosis, overcoming drug resistance, and immune escape is imperative. This will promote the development of precision medicine, thereby improving the patient’s prognosis.

**Fig. 1:**
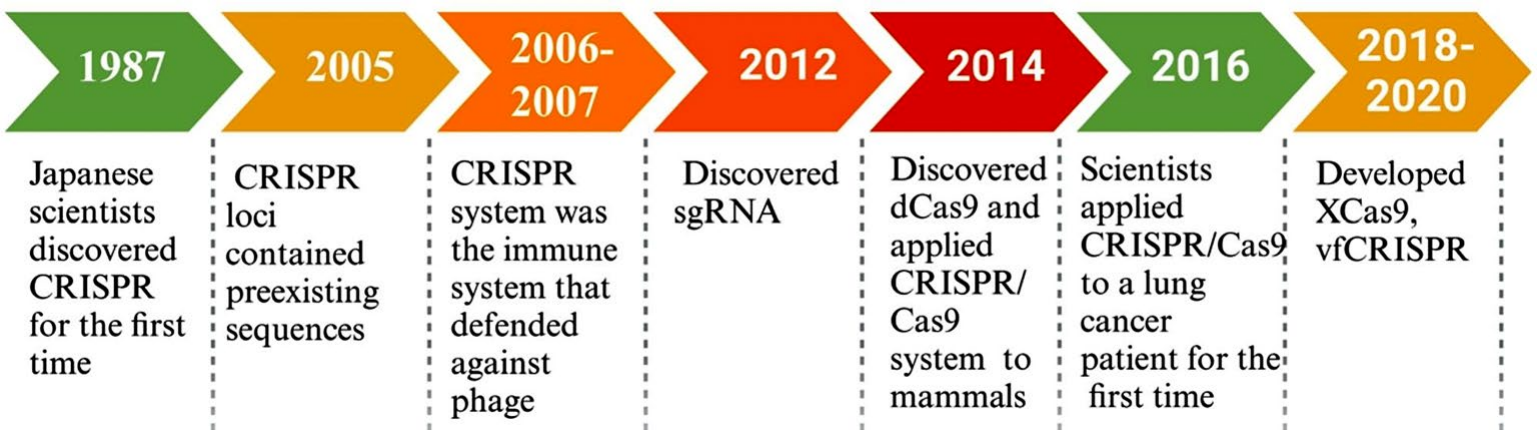
The brief development timeline of the CRISPR system. CRISPR array was firstly reported by Japanese scientists, and gradually developed into a versatile gene editing tool

An adaptive immune system of bacteria or archaea, the clustered regularly interspaced short palindromic repeat/CRISPR-associated nuclease 9 (CRISPR/Cas9) system was first discovered by Japanese scientists in 1987 [9]. Extensive research has led to the development of the CRISPR/Cas9 system into a powerful gene editing tool (Fig. 1) [9–18]. In fact, Jennifer Doudna et al. won the Nobel Prize in 2020 for their contribution to finding dual RNA-guide DNA editing [14]. CRISPR/Cas9 technique provides the advantage of rapidity, simplicity, and fidelity compared with other traditional gene editing tools, such as, zinc-finger nucleases (ZFN), transcription activator-like effector nucleases (TALENs), and pentatricopeptide repeat proteins (PPRs) [19, 20]. Moreover, a major breakthrough in CRISPR/Cas9-based screening library technology is the use of bioinformatics screening. It has been widely applied in cancer research, especially genome-wide screening in cell lines (2D), organoid models (3D), and mouse models [21, 22]. For example, Birgitta E et al. [22] using CRISPR/Cas9 screening in human colon organoids, identified TGF-β receptor 2 (TGFBR2) as a prevalent tumor suppressor gene (TSG) in CRC. Similarly, the tumor-suppressive SWI/SNF chromatin remodeling complex was found to be related to the TGF-β resistance [21]. Another potential target of wnt/β-catenin signaling in CRC, the histone lysine methyltransferase 2 A oncoprotein (KMT2A), has been identified by functional genomic screens as regulator of β-catenin transcriptional output [23]. CRISPR/Cas9 system also contributes to improving the effectiveness of CAR-T cell therapy. In 2021 a study by Dongrui et al., using genome-wide screening in CAR-T cells and glioblastoma stem cells (GSCs), found that the knockout (KO) of TLE4 and IKZF2 improved the anti-tumor efficacy of CAR-T cell therapy [24].

In this review, we discussed recent advances in CRC research with the help of CRISPR/Cas9 system, including mouse and organoid construction, genome-wide screening library, deeper investigation of lncRNAs, drug resistance genes, TSGs, hereditary CRC-related genes, genes related to immunotherapy, gene therapy and identification of novel players involved in inflammation, Lynch syndrome (LS), Colitis-associated cancer (CAC) and Inflammatory bowel disease (IBD). Specially, the limitations of CRISPR/Cas9 system, mainly related to the off-targeting, were discussed.

## The mechanism of the CRSPR/Cas9 gene editing

**Fig. 2:**
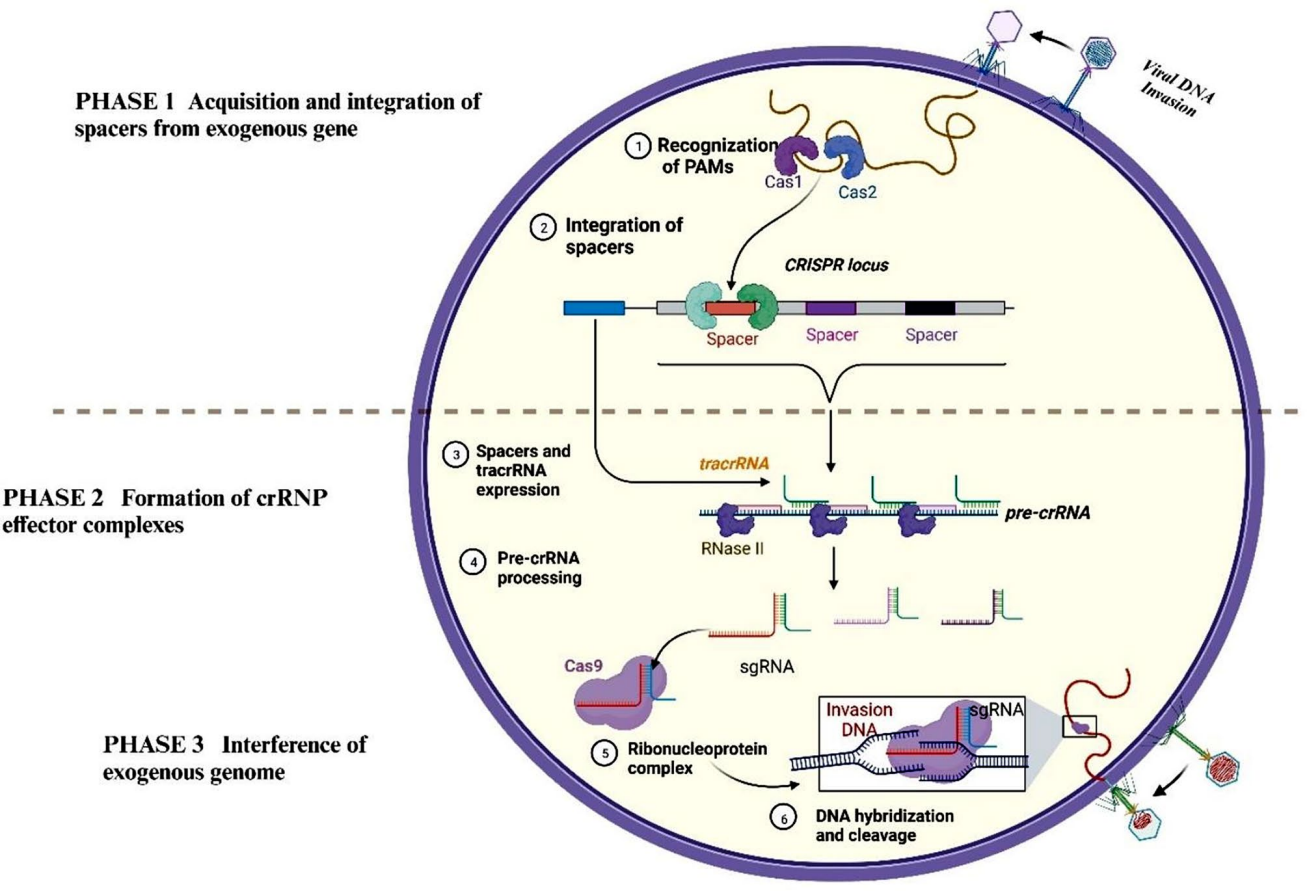
The mechanism of the CRISPR/Cas9: It is divided into three steps. First, when a viral DNA invades, CRISPR/Cas9 recognizes the PAMs of invaded DNA and cleaves them into suitable spacers, which are then selected and integrated into the CRISPR loci. Second, the CRISPR loci transcribe and form the effector complexes (crRNP) with the help of RNase III, Cas1/Cas2 complex, and other Cas proteins, tracRNA:crRNA structure in the type II system can be a single-guided RNA (sgRNA). Third, the Cas9 nuclease domain (including HNH and Ruvc) cleaves DNA through its nucleolytic activity, the HNH-like domain cleaves the DNA chain paired with crRNA, and the Ruvc-like nuclease domain cleaves another one

The CRISPR system is the adaptive immune system of bacteria or archaea that acts in defense against viral invasion [25]. CRISPR/Cas9 system includes CRISPR loci which consist of the diverse spacers (derived from phage, extrachromosomal elements, or preexisting sequences) and the interspaced short palindromic DNA repeats elements, along with the associated endonuclease 9 (Cas9) to defend against the foreign gene invasion [10, 26]. The division of CRISPR/Cas systems into three major types and several subtypes is based on the repetitive sequence and Cas gene or protein. This review is focused on the type II CRISPR/Cas system as it has the advantage of targeted gene editing. Gene editing by CRISPR/Cas system includes the following three steps (Fig. 2).

### The acquisition and integration of spacers from exogenous gene

In short, the CRISPR system of the host specifically recognizes the protospacer adjacent motifs (PAMs) of the exogenous gene after the plasmids or phages invade, and is cleaved into a suitable-size spacer sequence [27]. Then these spacer sequences are selected and integrated into the CRISPR array with the help of the Cas1/Cas2 protein complex and other Cas proteins, forming the flank array in the CRISPR array [28]. Thereby helping the creation of the specific genetic memory of infection in the CRISPR/Cas9 system.

### Formation of crRNA ribonucleoprotein (crRNP) effector complexes

CRISPR arrays transcribe into crRNA precursors (pre-crRNA), including repeat and spacer sequences. Meanwhile, trans-activating CRISPR RNA (tracrRNA) is formed by transcription of the trans-activating CRISPR RNA gene upstream of the CRISPR loci [29]. TracrRNA is complementary to pre-crRNA and functions in inducing the maturation of pre-crRNA and activating crRNA-guided DNA cleavage [14, 26]. In the type II CRISPR system, the maturation of pre-crRNAs requires a CRISPR array, Cas operon, as well as RNase III [30]. Processing by RNase III, a stable crRNP effector complex is formed by the association of the crRNA-tracrRNA complex with Cas9. Recent studies have shown that the tracRNA:crRNA structure can be a single-guided RNA (sgRNA) [14]. By designing an sgRNA sequence complementary to any target DNA sequence, Cas9 greatly simplifies the type II system and improves the efficiency and ease of gene editing.

### Interference of exogenous genome

The Cas9 nuclease in the Cas9-RNP complex specifically recognizes the PAMs of the invading DNA (as described before) and then binds to the spacer domain [31]. Firstly, a seed sequence is formed by a combination of the protospacer sequence with the 7–8 complementary base sequence of the spacer domain, which then expands outward and results in DNA strand replacement and R-loop structure that might activate the intrinsic nuclease activity. Finally, the DNA is cleaved by the Cas9 nuclease domain (including HNH and Ruvc) through its nucleolytic activity, the HNH-like domain cleaves the DNA strand paired with crRNA, and the Ruvc-like nuclease domain cleaves another strand [32, 33].

**Fig. 3:**
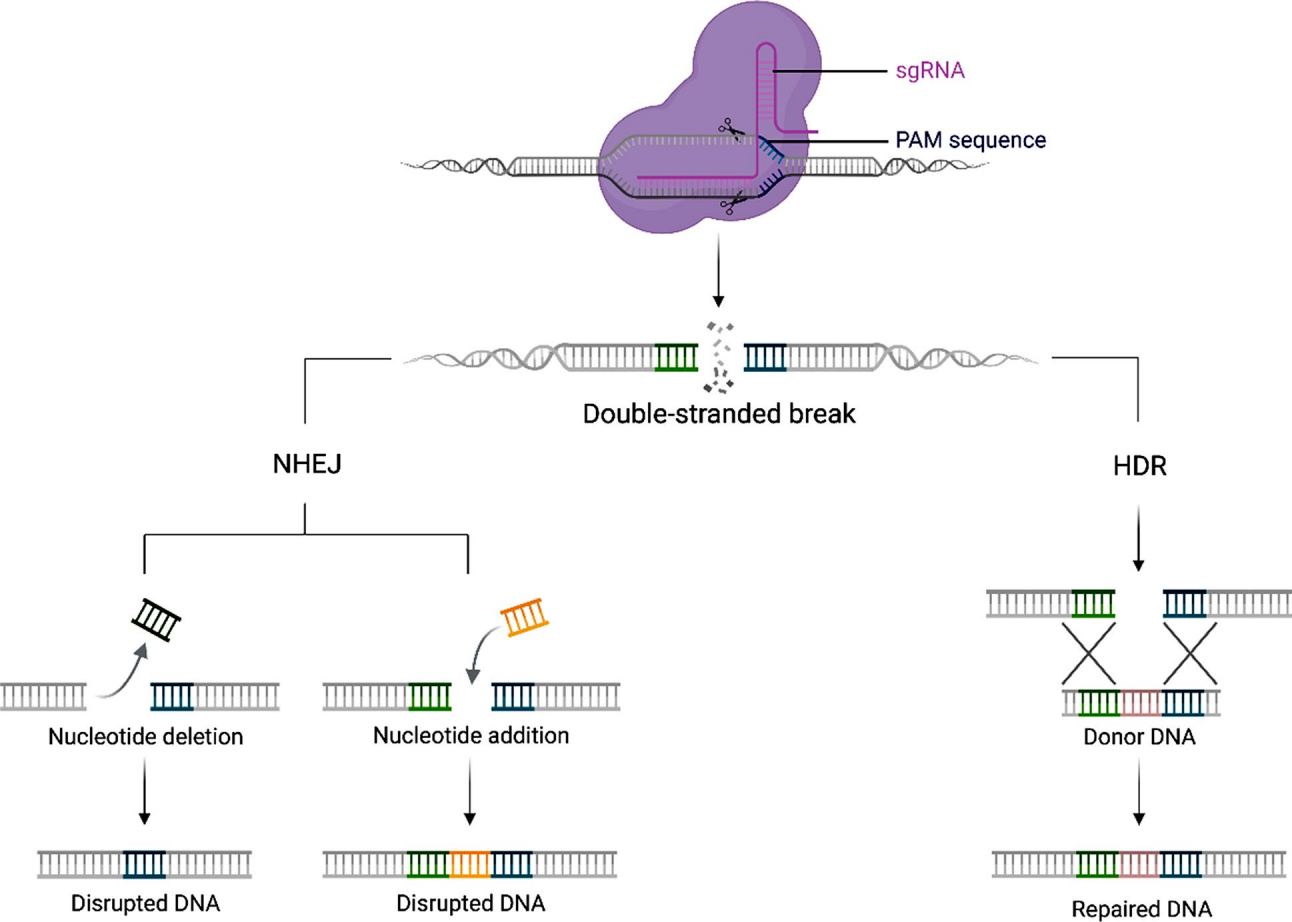
DNA damage repair system begins when viral DNA is cleaved. HDR harnesses the sister chromatid to repair the damage, achieving precise gene editing. On the contrary, the NHEJ pathway randomly repairs the DNA, causing disrupted DNA products. Both repair pathways can lead to gene mutations

Following the interference, DNA repair is done either by Nonhomologous end joining (NHEJ) or homology-directed repair (HDR) (Fig. 3). HDR uses the sister chromatid as a template for DNA repair and gives a precise product. On the contrary, NHEJ utilizes little or no sister chromatids and thus induces random gene editing products [34]. However, in mammals, the choice of DNA repair pathway is asymmetric. NHEJ is the dominant repair pathway as it is faster and more active than HDR. Nevertheless, NHEJ is an error-prone pathway, causing nucleotide insertions or indels, and can be utilized for gene editing (silence gene). On the contrary, HDR can be used for precise genome editing due to its high fidelity [35].

## Application of CRISPR/Cas9 system in CRC

**Fig. 4:**
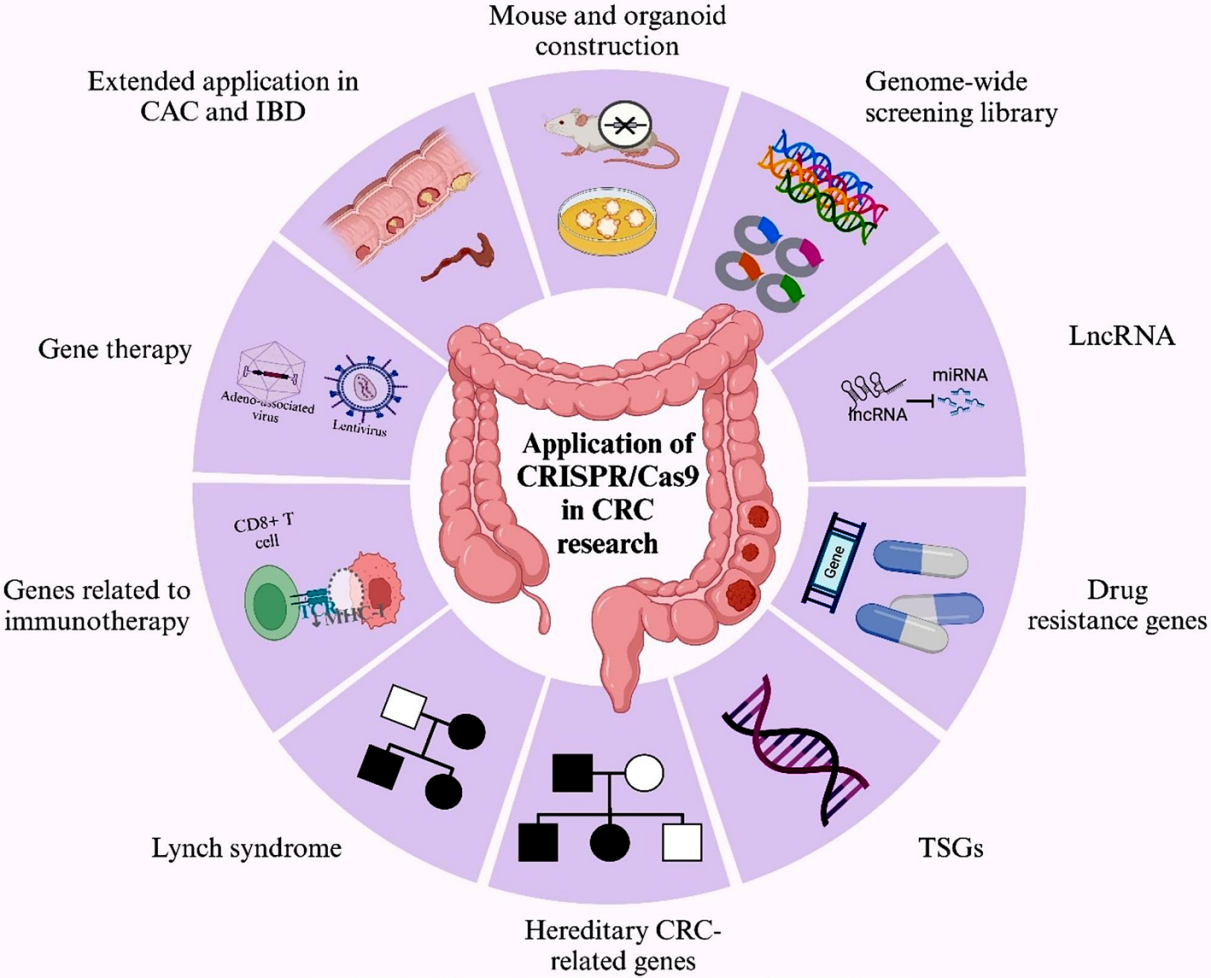
Current applications of the CRISPR/Cas9 system in CRC research, includes mouse and organoid construction, genome-wide screening library, deeper investigation of lncRNAs, drug resistance genes, TSGs, hereditary CRC-related genes, genes related to immunotherapy, gene therapy and identification of novel players involved in inflammation, LS, CAC and IBD

CRC evolution is significantly affected by the accumulation of gene mutations. However, the specific function of the genes and the impact of these genomic alterations is still elusive. Traditional gene editing tools possess many limitations, especially gene editing efficiency. Compared with other tools, CRISPR/Cas9-mediated genome editing is simple and effective. Additionally, precise genome editing can be achieved by using sgRNA of the CRISPR/Cas9 system. It has been used in CRC cell lines, mouse models, and human-derived organoid models, as well as CRISPR/Cas9-based gene screening and gene therapy. In the following, we will provide brief overviews of specific applications of the CRISPR/Cas9 system in CRC (Fig. 4).

### Mouse and organoid model generation

Animal models and 3D cell culture offer multiple advantages over the traditional 2D cell culture techniques for the study of cancer biology, such as, (a) Cells (cancer cell, epithelial cell, cancer-associated fibroblast (CAF), infiltrating immune cell, and others) interactions are more in accord with real cancer and environmental “niches” are created; (b) The cell features such as morphology and division mode with various phenotypes and polarity are closer to that in vivo; (c) Chemical gradients (oxygen, pH, metabolites, and growth factor gradients) are more in accord with real cancers; (d) Molecular mechanisms such as gene expression and splicing are closer to that in vivo, (e) The efficacy of antineoplastic drugs is better evaluated [36–39]. CRISPR/Cas9 technique has been successfully applied to plenty of mammals and tumor organoid models [40–44]. CRISPR/Cas9 system has helped in establishing specific gene KO human intestinal organoid and mouse, tumor models for CRC. Human intestinal organoid cells have been edited by CRISPR/Cas9 by designing specific sgRNA to introduce gene mutations, successfully harboring TSG mutations model, AKSTP (*APC, KRAS, SMAD4, TP53, PIK3CA*) organoid models. The organoid models were then cultured under different conditions. Subsequently, tumors were formed by implanting cells under the kidney subcapsular and spleen of mice. However, no metastasis was observed, indicating that the “drive” pathway mutation could survive in an unfavorable tumor microenvironment but could not metastasize [45]. In vitro, long-term cell line cultures are established by multiple mutation models, whereas in vivo cell cultures are dependent on the artificial addition of various cytokines. CRISPR/Cas9 was implemented to KO the common CRC mutant genes (*APC, TP53, KRAS, SMSD4*) in intestinal organoid stem cell lines that were independent of all stem cell growth factors in vitro to create a cell line model closer to the natural tumor environment and independent of growth factors. As a result, four mutated organoids grew into tumors with invasive characteristics in vitro [46]. In a study done by Jatin Roper et al. in 2017, they constructed a mouse colon tumor model by designing the APC and Trp53 genes sgRNA lentivirus site-directed mutation of TSG in mouse colon [47]. Subsequently, they successfully transplanted human CRC organoids into the colon mucosa of tumor-free mice using endoscopy. Viral infection of the mucous membrane was found to be limited to the transplant area via immunofluorescence tracing. Later, a colonoscopy showed that the mice developed tumors within six weeks, and characteristics of abnormal activation of human colon tumor and Wnt signal pathway were observed. Their study successfully simulated in vivo tumor formation and metastasis. Moreover, the clone formation of the leucine-rich repeat sequence, including G protein-coupled receptor 5 (Lgr5+) stem cells, was characterized through this model. Additionally, a platform for the functional verification of tumor-driving genes has been created in the form of the organoid model. Another study by Haruna Takeda et al. established a stable expressed Cas9 AK (*Apc, Kras* mutation) organoid model to verify the function of 29 TSGs [48]. In 2020, CRISPR/Cas9 technique was used to construct a human serrated adenoma model (TSAs) to explore CRC with RSPO fusion gene and GREM1 overexpression [49]. To date, CRISPR/Cas9 gene editing techniques have been combined with many kinds of organoids in order to investigate cancer and drug-resistance genes [50–55]. It overcomes the shortcomings of traditional cell line culture and better simulates the biological behavior of tumors in a complex environment when combined with directional mutation of CRISPR/Cas9 gene editing. The combination provides a powerful platform for tumor research (Table 1).

**Table 1:** The application of the CRISPR/Cas9 system in a mouse or organoid model

Model	Target gene	KO or Overexpression	Experimental data	Ref
Human intestinal organoids	AKSTP	KO	Survive in an unfavorable tumor microenvironment	[45]
Mouse colon tumor model	APC, Trp53	KO	Functional verification of tumor-driving genes	[47]
Organoids	TSGs	KO	Verify the function of 29 TSGs	[48]
Human serrated adenoma	RSPO fusion gene and GREM1	Overexpression	Harbor histopathology features of TSAs	[49]
Human colon organoids	APC	KO	Precise stratification of Wnt responses in CRC	[50]
Early-onset	APC	KO	Upregulate PTK7 protein and suppress BMP2	[53]
(sub-cutaneous and colon orthotopic) mouse models	MUC5AC	KO or Overexpression	MUC5AC KO reduces 5-FU and oxaliplatin resistance	[52]
intestinal organoid-based functional model	DACH1	Overexpression	Overexpression of DACH1 stimulates colony formation and tumor organoid formation	[51]
Nrp2- KO murine CRC organoids	Nrp2	KO	Maintaining the aggressive phenotype and survival of tumor-derived CRC organoids	[55]
Migration patterns and clonal origins	ZFP36L2	KO	Enhances the metastatic potential of CRC cells	[54]
Intestinal organoid stem cell model	APC, TP53, KRAS, SMSD4	KO	Construct a cell line free of all stem cell growth factors	[46]

### Genome-wide screening library based on CRISPR/Cas9 system

CRISPR/Cas9-mediated perturbations include the KO, interference (i) or activation (a) of a specific gene, or of a pool of genes targeted by a CRISPR/Cas9 KO, CRISPRi or CRISPRa libraries. The introduction of CRISPR pooled libraries, up to a genome-wide scale, recently applied in cancer research, represent the latest revolution of this system, since allows the functional investigation of hundreds or thousands of genes simultaneously [56–58]. CRISPR KO application is based on the typical gene manipulation mechanism of type II CRISPR/Cas9 system that generates double stranded DNA breakage (DSB) as described above, DNA was repaired by NHEJ or HDR, resulting frameshift, insertion, even introduced terminator causing premature stop codon. Thus, genome editing of CRISPR KO was one-off manipulation. Of note, there still exists other disadvantages such as off-target effects, low editing efficiency and unknown toxicity of DSB [59]. CRISPRa and CRISPRi harbored the similarity editing mechanism which achieved by dCas9. dCas9 was the variant of Cas9 that loss the function of HNH and Ruvc nucleolytic activity but its sgRNA target function. Both systems could regulate expression (activation or inhibition) of specific genes through transcriptional regulation. dCas9 was designed to target the transcriptional start site (TSS) or transcriptional factors, difference is that CRISPRa targeted transcriptional activators while CRISPRi targeted repressors. Typical examples were dCas9 fusion to transcriptional repressors: Kruppel-associated box (KRAB) domain, can significantly inhibit gene expression including noncoding genes (microRNA or large intergenic noncoding RNAs) and coding genes. On the contrary, dCas9 fusion to VP16、VP64 or other domains could activate gene function even microRNA and lncRNA [60–62]. Another discrepancy was targeted sequences requirements, although both should closer to TSS, CRISPRa required closer to TSS (-300 to 0) while CRISPRi ranged from − 50 to + 300. However, both CRISPRa and CRISPRi applications were limited since a gene can be regulated by multiple TSSs or multiple genes regulated by the same TSS wherein CRISPRa and CRISPRi introduced unidentified effects (additional genes expression or inhibition) [63]. In this section, we focus on CRISPR KO application in CRC due to the increased use in CRC research.

Lately, CRISPR/Cas9 KO library has been used to screen proto-oncogenes, TSGs, and tumor drug resistance genes and for the construction of tumor models in xenograft mice [22, 64–66]. KRAS mutations are common in CRC cases, accounting about 40% of all CRC patients, especially in the right-side CRC (appropriately 85%) [67]. Unfortunately, CRC patients with KRAS mutation are resistant to the first-line chemotherapy (FOLFOX) and anti-EGFR therapy, associates with a high recurrence rate [67–69]. Thus, it is urgent to make breakthrough in the potential mechanism of KRAS mutation in CRC. Genome-wide CRISPR has been adopted to screen the necessary regulatory factors for the growth of KRAS mutant CRCs. By constructing a CRC mouse xenograft model, harboring KRAS mutant, it was found that the chromatin remodeling protein (*INO80C*) acts as an inhibitory factor of KRAS mutant CRC [64]. In addition, synthetic lethal screening of KRAS mutant CRC was done using the CRISPR/Cas9 KO library technique. Researchers adopted the selective ploidy ablation that counterfeited cancer-specific gene expression changes in the yeast gene disruption library and identified IRE1 harbored synthetic lethal effect with RAS mutation in yeast. Despite genome-wide CRISPR/Cas9 screening in KRAS mutant CRC cells found that the human ortholog gene (ERN1) could not inhibit the proliferation, the KO of ERN1 rendered sensitivity to MEK inhibitor. Further genetic screening in ERN1- KO KRAS mutant CRC cells led to the identification of several negative regulators of JUN N-terminal kinase (JNK)/JUN signaling [70]. In subsequent research, GRB7 through the RTK pathway caused KRAS mutant colon cancer resistant to MEK inhibitors [71]. These findings revealed the specific resistance mechanism to MEK inhibitors and provided a new therapeutic target for KRAS mutant CRC.

CRISPR/Cas9 has also contributed to investigating the mechanism of the classical Wnt signaling pathway essential in maintaining tissue homeostasis and development [72]. Abnormal Wnt signal pathways can widely affect cell proliferation, differentiation, and tumorigenesis [73]. They can be divided into β-catenin-dependent classical pathways and non-classical pathways. Classical signaling pathways are mainly associated with CRCs. Despite the identification of many molecular transduction mechanisms of Wnt signaling pathways, targeted therapy remains a challenge [74]. Screening the regulatory network of classical Wnt/β-catenin signal transduction revealed the role of β-catenin in epigenetics. The potential of its transcriptional output KMT2A/mLL1 as an epigenetic regulator in targeted therapy was also identified in CRC [23]. In the same year, another study by Evron T et al. reported the potential key role of DExH-box protein 29 (DHX29) as a classical TSG in Wnt signal transduction. They utilized genomic CRISPR/Cas9 KO screening based on the Wnt signaling-induced cell survival. They demonstrated an inverse relationship between initiation factor DHX29 and cyclin-D1 in vitro, wherein knockdown and overexpression of DHX29, upregulated and inhibited cyclin-D1, respectively [75]. In recent in vivo studies, the targeting therapy of KRAS mutant CRC cells was combined with the classic inhibition of abnormal Wnt signaling activation. CRISPR/Cas9 screening of KRAS mutant CRC cells revealed that the activation of the Wnt signal was related to the expression of anti-apoptotic BCL-2 family genes in CRC cells. And the death of CRC cells was increased when BCL-X_L_ inhibitor (ABT-263) was added. Moreover, ABT-26 and Wnt signal inhibitors displayed synergistic effects. The study provided a new combined therapy strategy for KRAS mutant CRC. CRISPR/Cas9 screening systems have led to the recent discovery of many therapeutic targets and summarized in Table 2 [76–81].

**Table 2:** Genome-wide screening based on the CRISPR/Cas9 system in CRC

Screening object	Screening library /Gene panel size	Screening results	Function	Ref
KRAS mutant cells	Human GeCKO v2 A library	INO80C	Suppressors of KRAS mutant CRC	[64]
CRC allelic imbalance (AI)	1928 candidate genes in AI regions and 198 control genes	79 alleles, TP53	Loss of TP53 drives AI	[76]
DNA damage response (DDR) genes	GeCKOv2 library	43 DNA repair genes	Protect colorectal cancer cells against the platinum drug oxaliplatin	[65]
Synthetic lethal screening of KRAS mutant CRC	Human GeCKO v2 A library	ERN1	RAS synthesis lethal Regulation of sensitivity of MEK inhibitors	[70]
KO Screening	Human GeCKOv2 library	Krüppel-like factor 5 gene (KLF5)	Regulation of tumor cell proliferation	[77]
Organoid TSGs	Twenty-nine candidate CRC TSGs	Ten TSGs	Suppressors of CRC	[48]
Organoid TSGs	TSG sub-library	TGFBR2	Suppressors of CRC	[22]
Intestinal organoids	TSG sub-library	ARID1A SMARCA4	The negative regulation function of TGF-β	[21]
Genome-wide screening	Not available	TOP2A	Independent predictor of curative effect.	[66]
Targeted KO screening	Epi-drug library	METTL3	activating m6A-GLUT1-mTORC1 axis to promote CRC	[81]
KO Screening	Human GeCKOv2 library	DHX29	Tumor suppressor in Wnt signal transduction	[75]
KRAS mutant cell line	Human GeCKOv2 library	BCL-2	Synergistic effects of ABT-263 (inhibition of BCL-2) and NCB-0846 (inhibition of WNT signaling) in KRAS mutations	[147]
Organoid 441 epigenetic regulators	Epigenetic library	Zinc finger MYND type containing 8 (ZMYND8)	Regulate YAP-high intestinal cancer with metabolic vulnerability.	[79]
Genome-wide KO	Epigenetic library	KMT2A/mLL1	Epigenetic regulatory factor	[23]
Genome KO	TKOv1 library	LDL receptor transport gene	selective vulnerability of YM155	[80]
Genome-wide KO	GeCKOv2 library	GRB7	Drug resistance of MEK inhibitors	[71]

Organoid models based on CRISPR/Cas9 screening have also been achieved with the development of organoid culture technology. In 2019, CRISPR/Cas9 screening was conducted to verify the function of the introduced mutant genes in the AK (*APC, KRAS*) mutations intestinal tumor organoid model. AK organoids stably expressing Cas9 were constructed, and 29 candidate TSGs were selected for verification. The 29 candidate TSGs were divided into three groups, and two different gRNAs libraries were designed. gRNA pool was transferred into the AK-Cas9-like organoid using lentivirus. Mutant-like organoids were found to have stronger tumorigenicity and liver metastatic ability compared to parental organoids (only AK mutation). Ten TSGs (*Trp53, Smad4, Pten, Spen, Fbxw7, Acvr2a, Arid2, M113, Acvr1b, and Ror2*) were further identified. Moreover, the model helped in verifying the inhibitory effect of these genes and the function of promoting tumor metastasis [48]. Using a similar model and screening, another study by Birgitta E. Michels et al. identified the TGFBR2 as the most common TSG [22]. Additionally, CRISPR/Cas9 screening in the organoid model has also played a significant role in screening drug-resistance drivers. It was used to screen the whole genome of the human intestinal organoid model (APC mutation or wild type) to investigate the driving factors of TGF-β drug resistance. TGF-β resistance was more in the APC mutant organoid model, compared with the APC wild type. Further research showed that SWI/SNF chromatin remodeling complex was involved in APC mutant. Future studies will discover more and more tumor genes, TSGs, and drug-resistance genes [21]. Thereby, CRISPR/Cas9 screening technology will contribute to identifying suitable target genes, which may be used for target treatments of CRC (Table 2). However, current application mainly concentrated on CRISPR KO system, lacking CRISPRa and CRISPRi. Compared with CRISPR KO system, CRISPRa and CRISPRi harbored a lower off-target rate and toxicity of DNA interference, thus CRISPRa and CRISPRi may gain more precise outcomes [59, 60]. Of note, CRISPRa and CRISPRi can contribute to identifying the long noncoding RNAs (lncRNAs) loci and disclosing their potential role in cancers [56]. Off-target and unknown effects as described above exists and hope to further researched to overcome.

### CRISPR/Cas9 system identifies CRC targets

As a typical solid cancer, the evolution of CRC involves plenty of gene mutations. And the application of the CRISPR/Cas9 system can greatly contribute to investigating the function of those genes. In this section, we would focus on the lncRNAs, drug resistance genes (especially oncogenes), TSG, as well as hereditary CRC-related genes (Table 3), and introduced orderly.

**Table 3:** The CRISPR/Cas9 mediated genome editing in CRC

Target gene		KO or Overexpression	Experimental data	Ref
LncRNA	CYTOR	Overexpression	Promote the progress of tumor cells and xenograft of colorectal cancers in vitro	[83]
	CCAT2	KO	Promote cell proliferation and differentiation	[84]
	Long RNAs	CRISPR/Cas9-based RNA-tracking system	Cellular mechanisms to selectively export diverse classes of RNA	[85]
	SNHG15	KO or Overexpression	KO increases 5-FU sensitivity Overexpression is resistant to chemotherapy.	[86]
Oncogene	NSD2	KO	Inhibition of survivability, proliferation, migration, and invasion	[88]
	TIAM1	KO	Mediate drug resistance	[89]
	Rho	KO	Increased the radiosensitivity of CRC.	[90]
TSG	LACTB	KO	Anti-tumor effect of TP53 cells	[91]
	TGM2	KO	Inhibited CRC cell growth	[92]
Hereditary CRC	FAF1	KO	Increase the apoptosis resistance and proliferation of CRC cells	[94]
	CHEK2	KO	Regulator of DNA damage response	[95]
LS	MSH2	KO	Construct MMR-defective phenotype but MSI-H cancer	[97]
	MSH2	KO	Identify eight valuable MSH2 variants for LS	[98]
	CTCF	KO	A -35 kb enhancer that bound CTCF regulates MLH1 expression	[99]

Aberrant expression of lncRNAs has been associated with tumorigenesis and metastasis, as well as tumor stage. CYOTR, a novel identified lncRNA in recent years, was overexpressed in colon cancer and demonstrated to be an oncogene that promotes the epithelial-mesenchymal transition (EMT) phenotype and conferred colon cancer resistance to oxaliplatin [82]. And its precise mechanism was poorly understood. CRISPR/Cas9 contributed to identifying interaction sites of CYTOR that interact with NCL and Sam68 thereby activating the NF-κB pathway and EMT [83]. Similarly, a new mechanism of lncRNA and miRNA crosstalk was revealed when the KO of CCAT2 increased miR-145 content in HCT-116 cells meanwhile negatively regulating miR-21 and decreasing the proliferation and differentiation of HCT-116 cells [84]. In 2018, a CRISPR/Cas9-based RNA tracking system was employed to monitor the transmission of RNA in secretory vesicles to recipient cells to investigate the function of lncRNA. It was found that gRNAs containing secretory RNA output signals could be transferred from donor cells to recipient cells. Moreover, the mechanism of selective cell output of different kinds of RNA was demonstrated [85]. Additionally, regulating tumor drug resistance is also one of the functions of lncRNAs. For instance, lncRNA (SNHG15) has been shown to regulate the resistance of colorectal tumor cells to 5-FU. CRISPR/Cas9 mediated KO of SNHG15 improved the sensitivity of tumor cells to 5-FU in vivo and in vitro. In contrast, cancer cells overexpressing SNHG15 were better chemotherapy resistant [86].

In the past decade, even with the remarkable progress made in developing and applying tumor chemotherapy and targeted immunotherapy, the prognosis of patients with advanced CRC remains dismal. Drug resistance is the major obstacle to CRC drug therapy [7]. Therefore, the identification of new targets and potential hallmarks is important for overcoming drug resistance, predicting drug sensitivity, and selecting appropriate treatment options [87]. In a study done by Zhao et al. in 2021, CRISPR/Cas9 targeted KO of nuclear receptor-binding SET domain protein 2 (NSD2) inhibited not only the viability, proliferation, migration, and invasion of CRC cells but also inhibited the growth of CRC in the mouse model [88]. On the contrary, overexpressing NSD2 increased tumorigenicity, thus providing a robust marker validated in CRC that might act as a new therapeutic target. In vitro KO of one of Wnt signal-related genes, T-lymphoma invasion and metastasis-inducing protein (TIAM1), increased tumor 5-FU chemical sensitivity. However, no impairment in the growth of tumor cells was observed in the control group (only TIAM1- KO) in the mouse model. These findings indicated that high expression of TIAM1 was one of the causes of tumor drug resistance and might act as a potential therapeutic target [89]. CRISPR/Cas9 has also been helpful in the study of radiotherapy resistance. Liu et al. detected the expression of the Rho GTP enzyme in CRC cells following radiotherapy and used CRISPR/Cas9 to establish Rho gene KO cell line and zebrafish model [90]. RhoB expression KO has been shown to increase the radiosensitivity of CRC through Akt and FOXM1 pathways [90]. However, extensive research is needed to overcome drug resistance.

Another study has shown that Beta-lactamase-like (LACTB) played an inhibitory role in TP53 wild-type colorectal tumors. LACTB KO from HCT116 cells enhanced tumorigenicity, whereas its overexpression inhibited cell proliferation, migration, and EMT. Interestingly, LACTB only played an anticancer role in cells containing TP53, revealing the mechanism of p53 and LACTB [91]. Similarly, the inactivation of tumor suppressor p53 due to the direct binding of multifunctional transglutaminase 2 (TGM2) led to the tumor escaping from apoptosis induction [92]. High expression of TGM2 might be a target for tumor inhibition.

Hereditary CRCs account for about 12–35% of all CRCs, with only a small number of cases caused by high penetrance CRC genes; meanwhile, the potential germline causes are still unknown [93]. CRISPR/Cas9 system has also proved useful in revealing the mechanism of hereditary CRCs. To identify the germline mutation mechanism of hereditary CRCs, KO of Fas-related factor 1 (FAF1) was done in the whole-exome sequencing of 75 patients with unknown CRC species (discovery cohort) and patients from 473 families as the validation cohort. The protein variants encoding unstable FAF1 were found in both the discovery and the validation cohorts. Moreover, FAF1 gene KO increased the apoptosis resistance and proliferation of CRC cells [94]. Another study found that germline mutations in the DNA repair pathway were associated with familial CRC [95]. Meanwhile, CRISPR/Cas9 also contributed to the research on LS that preceded hereditary CRC. LS is an autosomal dominant disorder, which harbors a high risk of developing various cancers, including CRC, and endometrial cancer. The underlying molecular event of LS is that at least one of the pathogenic germline mutations in the DNA MMR genes (MSH2, MLH1, PMS2, and MSH6) or EPCAM gene leads to dMMR status [96]. However, a recent study reported that the MMR gene MSH2 mutation was not the only factor that leading to dMMR status in LS. Researchers introduced the typical alteration of LS (MSH2) into Hela cells by CRISPR/Cas9 system, despite the MMR-defective phenotype being constructed, engineered cells failed to exhibit MSI-H cancer [97].Thus, it is necessary to identify novel deleterious mutations in LS. Subsequently, Hayashida, Genki et al. [98] aimed to identify the function of the variants of uncertain significance (VUS) of LS. Site-specific MSH2 VUS of human embryonic stem cells were introduced by the CRISPR/Cas9 system, and eight potential pathogenic LS variants were identified. Another study focused on the MLH1 expression regulation and demonstrated that the expression of MHL1 was regulated by a -35 kb enhancer that bound CTCF (a protein with DNA-binding domain). The KO of the core region of CTCF by specific CRISPR/Cas9 decreased MLH1 expression [99].

These studies provide insights into the pathogenic mechanism of hereditary CRCs and may hopefully contribute in improving the accurate diagnosis, genetic counseling, and prevention of hereditary CRC.

### Application in CRC immunotherapy

Multiple pieces of evidence have shown that a high tumor mutation burden indicates an effective immunotherapy response, and immune checkpoint inhibitors (ICIs) can effectively treat metastatic colorectal cancer (mCRC) with low microsatellite instability and deficient mismatch repair. However, current ICIs are still ineffective for pMMR CRC or MSI-H CRC (known as pMMR-MSI-H tumors) [8]. Therefore, exploring new treatment strategies, rendering these tumors “immune-competent” and amenable to effective immunotherapy interventions, is a priority. CRC stem cells (CRCSCs) have the capability to escape the immune system and are immunotherapy resistant. Besides, high expression of programmed death-ligand 1(PD-L1) in CRCSCs promotes their stem-like properties and immune escape [100]. Therefore, understanding the mechanism of stem-like properties of CRCSCs and PD-L1 expression helps in developing new immune methods for CRC treatment.

In CRC, histone modifier AT-rich interaction domain-containing protein 3B (ARID3B) can regulate the expression of target genes, including intestinal stem cell (ISC) genes, Notch target genes, and PD-L1 genes. CRISPR/Cas9 mediated ARID3B KO in the xenografts (derived from CRC patients) revealed that ARID3B activated Notch target genes, ISC genes, and PD-L1 through recruiting lysine-specific demethylase 4 C (KDM4C) that modulates the chromatin configuration for transcriptional activation [101]. These findings explained the immune escape mechanism of CRCSCs and might provide a new immunotherapy method. Another study showed that CRISPR/Cas9 could efficiently target PD-1 in tumor-infiltrating lymphocytes (TIL) to produce adoptive T-cell therapy (ACT) products based on TIL-deficient PD-1 molecules. A sgRNA for TIL (from 5 patients with MSS or MSI CRCs) was designed to specifically KO the PD-1 of TIL. The efficiency of traditional PD-1 gene editing by ZFNs and TALENs was 76% and 72%, respectively. However, the gene editing efficiency significantly improved with the CRISPR/Cas9 reaching 87.53% [102]. This study highlighted the applicability of CRISPR/Cas9 to TIL-based ACT and provided a new method for immunotherapy, which can be extended to multi-gene editing ACT immunotherapy in the future.

### Gene therapy for CRC

Gene therapy has always been a popular subject. The metastatic site of CRC provides favorable conditions for targeted gene therapy as the sites are relatively limited to the intestinal cavity, liver, or abdomen, compared with other cancers such as breast cancer and lung cancer. Traditional gene therapy methods include gene modification or replacement, virus-directed enzyme-prodrug therapy (VDEPT), immune genetics, and oncolytic virotherapy (modified or natural virus that selectively targeted cancer cells without killing normal cell, for example, attenuated next generation oncolytic virus M1 (NGOVM), directed evolution on CRC cell line HCT116, achieving favorable oncolytic effect up to 9690 times) [103, 104]. However, no apparent curative effect of the clinical gene therapy of CRC has been reported to date. CRISPR/Cas9-based gene therapy is possible because of the precise editing by sgRNA-guided Cas9 nuclease. In 2020, researchers constructed the Cas9-GFP/sgRNA co-expression vector by synthesizing sgRNA partially complementary to the β-catenin Δ TCT (bases of RNA) ser45 deletion sequence. Later, in the HCT-116 cell line, the heterozygous TCT deletion mutation of β-catenin was corrected using the 96-nt single-stranded oligodeoxynucleotide (ssODN) of wild type β-catenin gene as HDR template. Interestingly with the correction of this mutation, the phosphorylation of β-catenin Ser45 was restored, and the activity of the classical wnt/β-catenin signal pathway was regulated, which could effectively inhibit the in vivo and in vitro proliferation of HCT116 cells [105]. In the same year, a team of Tao Wan et al. designed and synthesized a supramolecular polymer (CP/Ad-SS-GD, complexing disulfide-bridged biguanidyl adamantine) to mediate intracellular delivery of CRISPR/Cas9 RNP (ribonucleoprotein) [106]. Moreover, the polymer had a ~ 90% loading rate to Cas9 and sgRNA. The polymer delivery system was shown to significantly inhibit the proliferation of the KRAS mutant SW480 cell line and induce apoptosis of SW480 in vitro. Moreover, tumor growth and metastasis in xenograft mice were effectively inhibited after modifying this polymer with hyaluronic acid (HA). Besides, the nanocomposite had lower systemic toxicity. In another study, HA-decorated phenylboronic dendrimer (HAPD) was utilized to deliver Cas9 RNP to double-target mutant APC and KRAS. The interference rate of APC and KRAS genes in vivo was 17.7% and 19%, respectively. In comparison, the interference rate was 11.4% and 14.6% in xenograft mice, 12.3% and 17.2% in the liver metastasis CRC model, and 17.9% and 16.0% in the lung metastasis model, respectively. Systemic administration of this system has been proven to effectively inhibit the growth of CRC in xeno-transplanted mice and significantly prevent CRC-induced liver and lung metastasis induced [107]. Since tumorigenesis results from the accumulation of multiple gene mutations, further studies are required to identify more genes and delivery systems with low systemic toxicity.

### Extended application in inflammation-CRC

Intestinal microbiota, inflammation and IBD have been shown to be one of the risk factors for CRC [108]. In this section, we discussed the application of CRISPR/Cas9 in help of research in those risk factors predisposed CRC (Table 4). Intestinal microorganisms can relieve chronic inflammation and immune response, interfering with host metabolism and interacting with host epigenetics, thus promoting the occurrence and development of CRC. However, the pathogenesis of microflora-mediated chronic inflammation in CRC is not well characterized [109]. In a study, *Salmonella typhimurium* SL1314 was implemented to infect HCT116 cells to investigate the mechanism of intestinal bacteria causing CRC. Interestingly, a significant decrease in the total protein content of Wnt1 in host cells was reported. Surprisingly, this did not occur at the transcriptional level but directly at the protein level that is regulated by Salmonella. CRISPR/Cas9 mediated Wnt1 gene KO in the HCT116 cell line reduced Wnt1 protein expression, which in turn promoted the release of inflammatory cytokines such as IL-8, IL-6, and GM-CSF and protected cells from Salmonella invasion and reduced tumor cell migration and invasion ability in vitro. This study revealed the mechanism of intestinal flora regulating Wnt1 expression and possibly leading to CAC [110]. On the other hand, CRISPR/Cas9 mediated TP53 gene KO established a long-term inflammation model of colon cancer cells to evaluate the effect of chronic inflammation on P53 function. Prolonged chronic inflammation promoted the proliferation and migration of the TP53 KO cell line in vitro. However, after the removal of an inflammatory stimulus, it returned to an average level suggesting that sporadic tumors can be affected by chronic inflammation but no change in essence [108]. A previous study indicated that intestinal Na^+^/H^+^ exchanger isoform 8 (NHE8) plays a role in maintaining intestinal mucosal homeostasis. The loss of its expression leads to UC-like conditions in the intestinal mucosa [111]. Recently, Xu, Hua et al. uncovered a novel function of NHE8, showing that the loss of its expression promotes CAC. Specifically, the loss of NHE8 expression in HT29 cells through CRISPR/Cas9 resulted in increased colony formation units in vitro and heightened tumorigenesis in vivo [112]. Similarly, the loss of colonic SMAD4 expression in mice through CRISPR/Cas9 increased the expression of inflammatory mediators and initiated CAC in mice exposed to dextran sodium sulfate [113] (Table 4).

**Table 4:** Extended application of inflammation-CRC

Inflammation or CAC	Target	KO or Overexpression	Experimental data	Ref
CAC	Wnt1	KO	Promote secretion of inflammatory cytokines, prevent Salmonella invasion and reduced tumorigenesis	[110]
CAC	Tp53	KO	Chronic inflammation could promote the development of sporadic tumors with TP53 mutation	[108]
CAC	NHE8	KO	Promote colony formation in vitro and tumorigenesis in vivo	[112]
IBD	BCL-G	KO	Promote the secretion of inflammatory chemokines CCL5 while decreased CCL20	[118]
IBD	HLA-DRB5	KO	Inhibit the secretion of IL-1α induced by lipopolysaccharide	[119]
IBD	miR-125a	KO	Inhibit the expression of pro-inflammatory cytokines (IFN-γ, TNF-α and IL-17 A) in vitro and aggravate colitis in mice	[120]
IBD	Fungal strain	-	HD strains promote inflammation of IBD host and could be an individual UC signature	[121]
IBD	Bacteria	-	As a vector tool that achieving target therapy of IBD	[122]

IBD represents a group of chronic, persistent inflammatory ailments primarily affecting the colon and rectum. This group encompasses Crohn’s disease (CD) and ulcerative colitis (UC). It has been reported that the incidence of CRC among IBD patients can reach as high as 18% after 30 years [114]. Current research corroborates that persistent chronic inflammation within the colonic mucosa, coupled with the host’s immune response triggered by gut microbiota and their byproducts, are pivotal factors contributing to the progression from IBD to CAC [115]. On one hand, chronic inflammation induces DNA oxidative damage, instigating a series of genetic and epigenetic mutations (TP53, COX2 mutations, and CpG methylation), rendering IBD tumorigenic [116]. On the other hand, an imbalance in gut microbiota heightens the risk of CRC in IBD patients, especially in the presence of pks + E. coli. In vitro and in vivo experiments have demonstrated that exposure to pks + E. coli by colonic epithelial cells and in IBD mouse models results in the development of cancer [117]. Thus, delving into the underlying molecular events induced by these high-risk factors can be instrumental in averting the transition from IBD to CRC. Regarding inflammation and immune response, Woznicki, Jerzy A et al. [118] demonstrated that BCL-G, a member of the BCL-2 family, is non-essential for apoptosis in an IBD cell model. The KO of BCL-G through CRISPR/Cas9 heightened the secretion of inflammatory chemokines such as CCL5 while diminishing CCL20. Similarly, the KO of HLA-DRB5 through CRISPR/Cas9 revealed that HLA-DRB5 abolishes the secretion of IL-1α induced by lipopolysaccharide, shedding light on the mechanism through which intestinal bacteria trigger inflammation and fibrosis [119]. Another study showed that miR-125a regulates the immune response in IBD patients. Specifically, the targeted KO of miR-125a in IBD CD4 + T cells through CRISPR/Cas9 reduced the expression of pro-inflammatory cytokines such as IFN-γ, TNF-α, and IL-17 A, leading to more severe colitis in mice [120]. As for gut microbiota, Li XV, et al. [121] developed a platform that combined CRISPR/Cas9-based fungal strain editing to investigate IBD host-fungal interactions. Their findings revealed that high immune cell-damaging (HD) strains exacerbate inflammation in IBD hosts, offering insights into the unique features of UC in individuals and thereby providing new targets for IBD patients. Furthermore, bacteria editing via CRISPR/Cas9 can contribute to the therapy of IBD patients. Researchers devised a thiosulfate-responsive bacteria, allowing for controlled bacterial vectors that can accurately deliver drugs to IBD patients [122]. Collectively, the application of CRISPR/Cas9 has not only advanced our understanding of the molecular mechanisms underlying CAC but has also facilitated research into conditions that precede CAC, a critical endeavor for the monitoring and prevention of CRC formation.

## Challenge and progress of CRISPR/Cas9 system

As described above, CRISPR/Cas9 contributes to various aspects in CRC research. However, there still remains limited due to its disadvantages: off-targeting, limited delivery methods. In fact, CRISPR/Cas9 editing tool mainly aim to achieve precise target gene modification, delivery method or other factors, to some extent, refer to the factors that affect the gene editing efficiency and precision. Therefore, in this review, we incorporate them into off-targeting and supplementarily summarize below.

### Off-targeting

CRISPR/Cas9 can not only edit targeting gene, but also along with unexpected large number of single-nucleotide variants in vivo [123]. PAM spacer sequence was recognized by CRISPR loci and transcribed to form sgRNA, and sgRNA can specifically lead Cas9 nuclease to any genomic array that matches PAMs. As the result, those genomic sequences which are similar to PAMs are prone to be wrongly recognized and cleaved, causing off-targeting. Besides, CRISPR/Cas9 system derives from bacteria and as the adaptive immune system to defend against phage invasion. Since evolution, Cas nucleases develop to “expand recognition” ability as a result of some mutations in phages which render escaping CRISPR system. Thus, those homology sequences that show certain degree similarity of PAMs will also be targeted by Cas nuclease.

### Progress of overcoming off-targeting

At present, successful methods that aim to overcome the off-targeting problem mainly grouped into four aspects; (1). Computational method; (2). CRISPR/Cas9 engineering (Cas9 and gRNA modification); (3). Advanced delivery method (for review see [124]); (4). Anti-CRISPR proteins using (for review see [125, 126]).

### Computational method

One of the major off-target factors is the unreasonable gRNA design and selection, previous evidences have demonstrated that reasonable sgRNA can significantly reduce off-target rate and minimize unexpectable cleavages and their products [127]. For example, 5’-end nucleotides are essential for the activity of gRNA, truncated gRNAs that complement less target regions (length within 20 nucleotides), spectacularly decrease the unexpectable mutations of off-target sites more than 5000-fold and harbor more specificity [128]. Another evidence validated that partial sequence of gRNA can be replaced by DNA, thereby decreased off-target editing, such as replacement of 3’ end crRNA [129]. On the other hand, off-target sites and off-targeting events prediction are also necessary to help researches to avoid undesired outcomes following cleavage. In recent years, more and more online tools contribute to designing gRNA and predicting off-targeting events, dramatically improving the fidelity and efficiency of CRISPR/Cas9 system in cell lines or human species (Table 5). Thus, with the help of those tools, CRISPR/Cas9 genome editing and genome-wide library screening in CRC will obtain more accurate results even successfully adopt to human gene therapy.

**Table 5:** Online gRNA design and off-targeting prediction tools

Tool	Description	Input	Output	Web site	Ref
CRISPOR	gRNA or pre-gRNA selection and expression	DNA sequence, genome or PAMs	PAMs and cleavage sites; Guide sequence + PAM + restriction + enzymes + Variants; Specificity score; Efficiency score; out-of-frame score; off-target mismatch counts; Possible off-targets locations, gRNA or off-target primer	http://crispor.tefor.net/	[148]
GenScript	Online sgRNA design tool (only spCas9)	Gene symbol or ID	sgRNA; PAMs; Location; On-target and off-target even overall score; Primer design	https://genscript.com/tools/gRNA-design-tool	[149]
CHOPCHOP v3	sgRNA design and post cleavage prediction	Gene/transcript number; genomic coordinates; sequences	genomic coordinates; Target sequence, sgRNA; Self-complementarity; GC content; Efficiency; Off-targeting; Primer; Cas9 nickases; Repair profile prediction	http://chopchop.cbu.uib.no/	[150]
Cas-OFFinder/ Cas-Designer/ Cas-Analyzer	sgRNA design, potential off-target and microhomology predictor	Target, sgRNA or PAM sequence, species	sgRNA; target sites based on microhomology prediction; off-target; genome-wide gRNA selection; HDR/NHEJ frequency	http://rgenome.net/cas-offinder/ http://rgenome.net/cas-designer/ http://rgenome.net/cas-database/	[151–153]
CRISPR MultiTargeter	sgRNA design for similar DNA sequences	Multiple sequences in FASTA format or identifier/s	Common or unique target; ActivityScore (only type II)	http://multicrispr.net/index.html	[154]
WU-CRISPR	Genome-wide CRISPR/Cas9 gRNA design	Gene Symbol; NCBI Gene ID; GenBank Accession	gRNA sequence; potency score; off-target status and analysis;	http://crisprdb.org/wu-crispr/	[155]
CRISPResso2	Base editing analysis (amplicon sequencing)	FASTQ sequence; amplicon sequence; sgRNA sequence; editing tool specification	Indel rates and nucleotide frequencies; NHEJ/HDR frequency; Multiple alleles; Primer editors; Base editors; Batch mode	http://crispresso2.pinellolab.org	[156]
CRISPR-ERA	gRNA design for ERA (editing, repression, activation) and genome-wide (CRISPR, CRISPRI, CRISPRa) sgRNA design	Gene name, sequence or location	sgRNA sequence; Distance to TSS; Target chromosome; first site sgRNA sequence location; Offset distance; Specificity, efficacy score and E + S score	http://CRISPR-ERA.stanford.edu.	[150]
E-CRISP	Binding sites finding and gRNA target annotation	Organism; Target sequence (gene symbol, ensembl ID,or FASTA); Design aim (KO, N/C-Terminal tagging, CRISPRi and CRISPRa);	sgRNA and target sequence; SAE Score (S: Specificity score A: Annotation score E: Efficiency score); Match String; percent of total transcripts hit	http://e-crisp.org/	[157]
CRISPRseek	Bioconductor package for sgRNA design and off-target analysis	Sequence/s	gRNA; off-target sequence; Targeting score and diff score	http://bioconductor.org	[158]
COSMID	Off-target sites analysis (insertions, mismatches, deletions)	gRNA sequence and parameters	Off-targeting include insertions, mismatches, deletions and mismatch score; Suitable primers	http://crispr.bme.gatech.edu	[159]
sgRNAcas9/ CRISPR-offinder/ CRISPRamplicon/ CRISPR-lib/ sRNAPrimerDB	Softwares to design sgRNA and predict off-target sites	Target sequence, genome sequence (FASTA) and default parameters	sgRNA; CRISRP target sequence; GC content; Annotation of off-targets with number of mismatches; Annotation of off-targets with 12-nt seed region of the sgRNA; Risk-evaluation	http://biootools.com	[160–164]
TIDE/TIDER	R package to verify a pool cells mutation following editing	Targeted pool of cells and sgRNA sequence	Major mutations of prediction editing sites; Mutation frequency	http://tide.nki.nl	[159]
CRISPy-web	Microbial genome sgRNA design	antiSMASH	gRNA; off-target site	http://crispy.secondarymetabolites.org/	[166, 167]
CRISPick	Selection sgRNA of multiple genomes, nucleases that include CRISPRi/a/ko	DNA sequences, FASTA files, transcript/gene IDs, Gene symbols, or coordinates	Candidate sgRNA sequences; PAM Policy (only NGG); CFD score (Cutting Frequency Determination); Off-target information (Tier and match bin policy); TSS position	https://portals.broadinstitute.org/gpp/public/analysis-tools/sgrna-design	[168, 169]
sgRNA Scorer 2.0	Multiple CRISPR system sgRNA activity prediction	Sequences and CRISPR system with parameters (PAM and spacer length)	specificity and activity of putative sites based on vector machine model	https://crispr.med.harvard.edu/sgRNAScorerV2	[170]

### CRISPR/Cas9 engineering

**Fig. 5:**
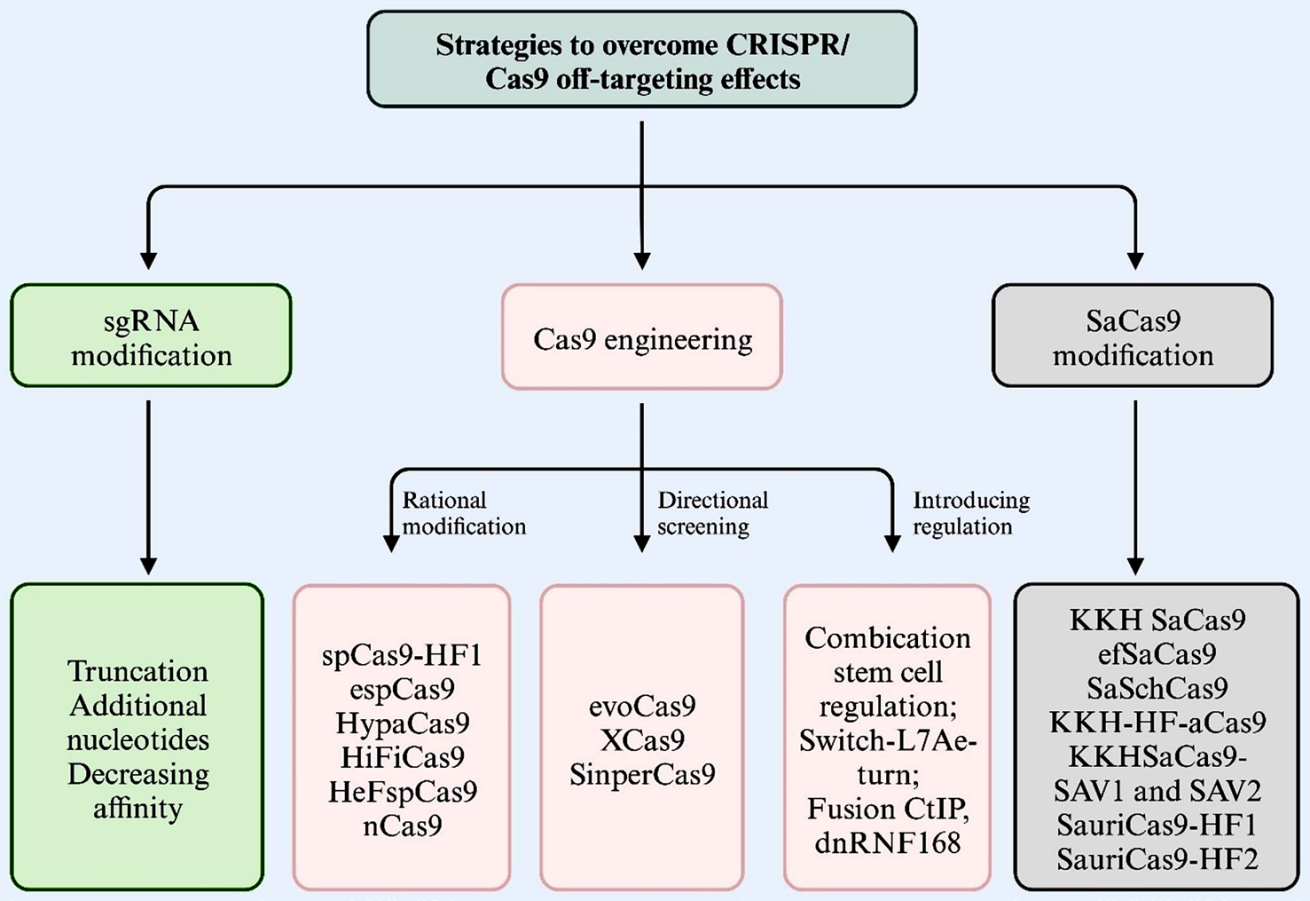
Summary of CRISPR/Cas9 off-target engineering, which divided into three sections: sgRNA modification, Cas9 engineering and SaCas9 modification

In this section, we will introduce the CRISPR/Cas9 engineering which improve the fidelity and specificity, mainly grouped into three categories: Cas9 engineering; sgRNA modification; SaCas9 modification (Fig. 5). Cas9 engineering could further divided into three methods: Rational modification (spCas9-HF1, espCas9, HypaCas9, HiFiCas9, HeFspCas9); Directional screening (evoCas9, XCas9, SinperCas9) (those two Cas9 engineering and sgRNA modification methods have been summarized, for review see [124, 130], we add another or new researches in this review); Introducing regulation.

Rational modification: DSBs were generated following Cas9 nuclease cleavage that guided by sgRNA, Cas9 nuclease domain (including HNH and Ruvc) cleaves the DNA strand paired with crRNA, and the Ruvc-like nuclease domain cleaves another strand [32, 33]. However, that’s the limitation that DNA generate two breaks, causing toxic lesions, unexpectable mutations, even leading cell death [131]. Recently, alternative DNA strand cleavage caught attention, which utilizes variant Cas nuclease, called Cas9 nickase (nCas9), including two types: D10 and H840A. Cas9 modification, especially Ruvc and HNH domains engineering (alanine substitutions) creates defective nucleases that just cleavages one DNA strand (D10 and H840A cleave gRNA target sequence and non-target strand, respectively), thereby reducing off-targeting [132, 133]. However, new evidence demonstrated that H840A can also create DSBs, although number of DSBs less than wild-type Cas9. To make H840A more specificity, researchers adopted double mutations variant (H840A + N863A) of Cas9 HNH domain showed no DSBs in vitro and achieved more accurate base editing outcome [134].

Introducing regulation: High activity of Cas9 nuclease can extra cleave target sites that imperfect guided by sgRNA [135]. For example, Cas9 cleavages occur not only to optimum PAMs (5-NGG-3), but also to 5-NGA-3 PAMs or target sequence that harbors 5-NAG-3 [136]. Expression regulation of Cas9 nuclease dedicated to limiting Cas9 activity thereby decrease extra cleavages. Combination transcriptional and protein regulations in human stem cell to limit Cas9 nuclease baseline expression and exposure time, observing a lower off-target rate but an efficiency on-target cleavage [137]. Or introducing a “self-regulates switch”, L7Ae:K-turn, to control Cas9 expression: Cas9 expression can be detected within 6 h and reduced through 72 h, thereby minimizing the chance of off-target [138]. Another strategy is that making DSBs repair prone to choose HDR, more accurate than NHEJ, achieved by fusion CtIP (CtBP-interacting protein) and dnRNF168 (dominant negative RNF168) to Cas9, decreasing off-target rate up to 7-fold while less genotoxic in human cells [139].

Sacas9 modification: SaCas9, refers to the Cas9 orthologues, derived from Staphylococcus aureus, characterized a larger targeting range, higher cleavage activity, and small enough to be integrated into adeno- associated viral (AAV) thereby delivered to cells [140, 141]. Thus, Sacas9 may display bright application prospects in biology research and human gene therapy. Indeed, directional screening of SaCas9 strikingly expands the targeting range, KKH SaCas9, a variant of SaCas9, demonstrated higher targeting activity than SaCas9 and up to 4-fold broad targeting range and approximate off-target events [140]. Besides, high-fidelity SaCas9 variant was also generated, efSaCas9 (enhanced-fidelity SaCas9, N260D mutation), which can discriminate single base mismatches, and dramatically decrease the off-target events, even up to 93-fold increase [142]. However, multiple PAM sequences recognition limits their application in human cell, the PAMs NNG-RRT, -RC, -RRR, -A, and -R can be recognized, and simple PAMs recognition seems particularly important. SaSchCas9, a new variant of SaCas9 can deal with this, simple PAM, NGGR recognition harbors parallel editing effect [143]. On the other hand, Cas9 engineering can also extend to SaCas9, SaCas9-HF, expands genome-wide specificity, considering the advantage of KKH SaCas9, KKH-HF-SaCas9 emerged as the times require. GUIDE-seq validated KKH-HF-SaCas9 remarkably increase the editing efficiency in human cells [144]. In order to achieve higher fidelity, scientists created KKHSaCas9-SAV1 and SAV2, integrating the advantages of KKH and HF variants, harboring new mutation (Y239H) on REC domain of KKH-SaCas9. Improved KKHSaCas9-SAV1 and SAV2, with base-pair distinguish, high targeting range and fidelity, could get closer to application in cancer gene therapy [145]. A newest ortholog, SauriCas9, scientists separated from Staphylococcus auricularis may offer a new option of gene editing tool. They assumed that the mutation of efSaCas9 can also fit in SauriCas9, and the outcomes, SauriCas9- HF1, SauriCas9- HF2 (N269D, N270N mutation, respectively), greatly increased targeting specificity, especially SauriCas9- HF2, even up to 111.9-fold improvements compared with primary SauriCas9 [146].

However, we observe that most modification of Cas9 are mutually independent, we hope that scientists can integrate all the advantages into one Cas9 variant, seem as the SaCas9 if possible. Besides, computational method helps us to design suitable sgRNA and could be modified. On the other hand, anti-CRISPR proteins also be adopted when gene editing in vivo or vitro. In summary, future research could concentrate on integrating all advantages that helpful to achieve higher fidelity and lower off-targeting, thereby the most precise gene editing will occur and applied in CRC research and therapy.

## Conclusion

CRC is highly malignant, especially the mCRC, with limited treatment options. The CRISPR/Cas9 system is a robust gene editing technology. CRISPR/Cas9-based genome-wide screening library and organoid models provide an efficient platform to study cancer biology and for making significant progress both in targeted therapy and overcoming drug resistance. This review summarizes different aspects of its applications in CRC research and discusses the challenge and chance of CRISPR/Cas9 application, providing insight into CRC biology and treatment options. CRISPR/Cas9 gene editing technology provides a new method for both targeted therapy and gene therapy. However, due to the off-targeting, and limited application of the CRISPR/Cas9 gene editing technology to cell lines and organoid models, further research is needed for gene therapy of CRC patients.

## Data Availability

All data and materials are included in the references.

## Abbreviations

CRISPR/Cas9: The clustered regulatory interspaced short palindromic repeat/CRISPR-associate nuclease 9
TSGs: Tumor suppressor genes
CRC: Colorectal cancer
PD-1/PD-L1: Programmed cell death 1/Programmed cell death ligand 1
CAR-T: Adoptive chimeric antigen receptor T
MSI: Microsatellite instability
pMMR: Proficient mismatch repair
ZFN: Zinc-finger nucleases
TALENs: Transcription activator-like effector nucleases
PPRs: Pentatricopeptide repeat proteins
KMT2A: Histone lysine methyltransferase 2 A oncoprotein
GSCs: Glioblastoma stem cells
PACE: Phage-Assisted Continuous Evolution
vfCRISPR: Very fast CRISPR
PAMs: Protospacer adjacent motifs
tracrRNA: Trans-activating CRISPR RNA
crRNA: CRISPR RNA
crRNP: crRNA ribonucleoprotein
sgRNA: single-guided RNA
NHEJ: Nonhomologous end joining
HDR: Homology-directed repair
CRISPRa/CRISPRi: CRISPR gene activation/interference
INO80C: Chromatin remodeling protein
DHX29: DExH-box protein 29
AK: APC, KRAS mutations
TGFBR2: TGF-β receptor 2
CAF: Cancer-associated fibroblast
Lgr5+: G protein-coupled receptor 5
TSA: Serrated adenoma
lncRNAs: Long non-coding RNAs
NSD2: Nuclear receptor-binding SET domain protein 2
TIAM1: T-lymphoma invasion and metastasis-inducing protein
LACTB: Beta-lactamase-like
EMT: Epithelial-mesenchymal transition
FAF1: Fas-related factor 1
KO: Knockout
LS: Lynch syndrome
VUS: Variants of uncertain significance
ICIs: Immune checkpoint inhibitors
mCRC: Metastatic colorectal cancer
CRCSCs: CRC stem cells
ARID3B: Histone modifier AT-rich interaction domain-containing protein 3B
ISC: Intestinal stem cell
KDM4C: Lysine-specific demethylase 4 C
TIL: Tumor-infiltrating lymphocytes
ACT: Adoptive T-cell therapy
NGOVM: Next generation oncolytic virus M1
VDEPT: Virus-directed enzyme-prodrug therapy
ssODN: Oligodeoxynucleotide
CP/Ad-SS-GD: Complexing disulfide-bridged biguanidyl adamantine
HA: Hyaluronic acid
HAPD: HA-decorated phenylboronic dendrimer
CAC: Colitis-associated cancer
IBD: Inflammatory bowel disease
NHE8: Na^+^/H^+^ exchanger isoform 8
CD: Crohn’s disease
UC: Ulcerative colitis
HD: High immune cell-damaging
nCas9: Cas9 nickase
